# LTDpathies: a Novel Clinical Concept

**DOI:** 10.1007/s12311-021-01259-2

**Published:** 2021-03-22

**Authors:** Hiroshi Mitoma, Jerome Honnorat, Kazuhiko Yamaguchi, Mario Manto

**Affiliations:** 1 Department of Medical Education, Tokyo Medical University, Tokyo, Japan; 2 French Reference Center on Paraneoplastic Neurological Syndromes, Hospices Civils de Lyon, Hôpital Neurologique, 69677 Bron, France; 3 Institut NeuroMyoGene INSERM U1217/CNRS UMR 5310, Université de Lyon, Université Claude Bernard Lyon 1, 69372 Lyon, France; 4 Department of Ultrastructural Research, National Institute of Neuroscience, National Center of Neurology and Psychiatry, Tokyo, Japan; 5 Unité des Ataxies Cérébelleuses, Service de Neurologie, Médiathèque Jean Jacquy, CHU-Charleroi, 6000 Charleroi, Belgium; 6 Service des Neurosciences, University of Mons, 7000 Mons, Belgium

In the cerebellar circuitry, the combined activation of parallel fibers (PFs) and climbing fiber (CF) results in long-term depression (LTD) of the excitatory synapses between PF-Purkinje cells (PC) [1–3]. Based on the finding that CF fire at high probability in motor failure [4], it is assumed that CF carry error signals related to motor performance and that PF-PC LTD is a fundamental mechanism of motor learning [5]. Together with previous theoretical studies by Marr and Albus [6, 7], the term Marr-Albus-Ito theory was coined for this hypothesis. However, the theory has been challenged by several groups during the last four decades. First, several other hypotheses have been proposed on the function of the CF-PC synapses and described by some as the “complex spike wars” [8]. Others have provided evidence against the view that CF encode feedback error signals [9]. Second, the interaction among various forms of synaptic plasticity challenges the view that single plasticity underlies a particular learning type [10]. Thus, others argue that the divergent forms of plasticity in the cerebellar cortex cooperate synergistically to ultimately create optimal output for any behavior [10–12].

Apart from the historical controversy, ataxic symptoms in some patients with immune-mediated cerebellar ataxias (IMCAs) have been attributed to dysregulated PF-PC LTD [13]. Here, we present the novel clinical concept of “LTDpathies,” a novel group of disorders targeting LTD.

In IMCAs, various synaptic proteins, such as glutamic acid decarboxylase 65 (GAD65), voltage-gated Ca channel (VGCC), metabotropic glutamate receptor type 1 (mGluR1), and glutamate receptor delta (GluR delta), are targets of auto-immunity [14–16]. Although the auto-immunity and clinical profiles vary enormously among CAs associated with anti-VGCC, anti-mGluR1, and anti-GluR delta antibodies (Abs), these three subtypes show the common clinical features of good prognosis with no or mild cerebellar atrophy in non-paraneoplastic syndrome [13, 17], suggesting functional cerebellar disorders without or with weak neuronal death. Notably, anti-VGCC, anti-mGluR1, and anti-GluR delta Abs are preferentially found in IMCAs and not in other autoimmune neurological conditions such as autoimmune limbic encephalitis [17]. This preferential distribution might reflect vulnerability in the functional roles of these auto-antigens among elementary cerebellar functions. VGCC, mGLuR1, and GluR delta are all involved in molecular cascades that serve to induce PF-PC LTD: VGCC and mGluR1 are molecules required for any increase in Ca^2+^ concentration and subsequent induction of PF-PC LTD [18–21], while GluR delta plays an essential role in AMPA receptor trafficking [22]. Consistently, IgG obtained from some patients with these subtypes blocked PF-PC LTD induction and impaired adaptation functions [23].

These studies suggest that anti-VGCC, anti-mGluR1, and anti-GluR delta Abs-associated cerebellar ataxias share one common pathophysiological mechanism, the deregulated PF-PC LTD. We propose that these three subtypes can be included into the novel entity of “LTDpathies,” which describes PF-PC LTD-related elementary dysfunction [13].

It should be acknowledged that CA in these IMCA subtypes cannot be exclusively attributed to deficits in PF-PC LTD. Basal synaptic transmission is impaired not only by anti-VGCC Abs but also by anti-mGluR1 and GluR delta Abs [24, 25]. The impairment in basal synaptic transmission will be layered by dysregulated PF-PC LTD, thereby amplifying functional disorders. However, to establish the clinical category of “LTDpathies,” the following questions about LTD-related elementary function should be answered.

First, does dysregulated LTD cause impairment in the online predictive control? Using the method of conditional knockout mice (a tetracycline-controlled gene expression system), one study reported that acute blockade of mGluR was associated with impairment of PF-PC LTD and simultaneous motor incoordination without affecting basal synaptic transmission [25]. The phasic suppression on PCs (i.e., disinhibition on dentate nucleus neurons with facilitatory output to the cerebral cortex) is assumed to be facilitated by PF-PC LTD-induced attenuation of PF excitatory inputs [26]. Although these studies suggest the involvement of PF-PC LTD in online predictive control, further evidence needs to be demonstrated.

Second, can dysregulated PF-PC LTD cause motor learning-related symptoms? Historically, the significance of PF-PC LTD has been considered solely in terms of motor learning, such as the vestibulo-ocular reflex and eyeblink conditioning. In addition, synaptic plasticity also plays a critical role in the capacity for restoration and compensation to pathologies, defined as cerebellar reserve [27]. In this regard, the presence of dysregulated PF-PC LTD can amplify any cerebellar cortex impairment (e.g., disorganized basal synaptic transmission). These assumptions should be examined in controlled experimental studies.

Third, is dysregulated LTD present in other etiologies, including degenerative cerebellar ataxias? If VGCC, mGluR1, and GluR delta are critical molecules that can induce loss of LTD-related elementary functions in CAs, one can assume that similar pathologies can also occur in degenerative diseases associated with disturbance of the same molecules. In this regard, the role of mGluR1 in the degenerative cascades seen in SCA1, 3, and 5 has already been reported [28]. In an animal model of SCA6 expressing appropriate pathological alleles within a CACNA1A protein, a VGCC subunit α1A, CF-excitatory postsynaptic currents were altered, suggesting dysregulated functions in the olivocerebellar system [29].

The concept of “LTDpathies” can have practical benefits to the management of CA patients. CA associated with anti-VGCC, anti-mGluR1, and anti-GluR delta Abs commonly responds well to immunotherapies, an outstanding feature in IMCAs [13, 17]. Thus, the new concept provides a warning for careful differential diagnosis in order to avoid the loss of therapeutic opportunities.

Furthermore, the introduction of the “LTDpathies” concept can provide new directions in physiological studies on PF-PC LTD. Disorganized prediction and damaged reserve might be related to impairment in execution and repair of the internal model, embedded in the cerebellum, respectively (Fig. 1) [13].

**Fig. 1:**
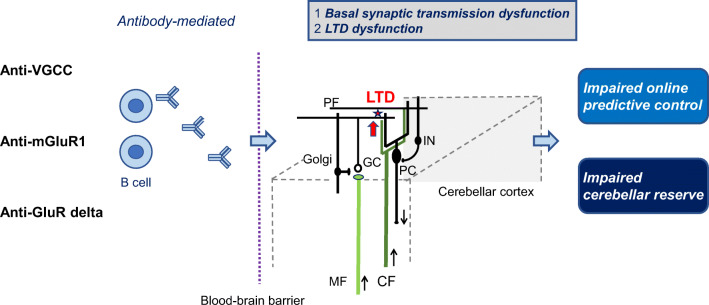
Schematic diagram of the pathophysiological mechanisms underlying anti-VGCC, anti-mGluR1, and anti-GluR delta-associated cerebellar ataxias (“LTDpathies”). The antibodies include dysfunction of basal synaptic transmissions and long-term depression (LTD), leading to impairments of online predictive control and cerebellar reserve. *PC* Purkinje cell, *GC* granule cell, *IN* inhibitory interneurons, *CF* climbing fiber, *PF* parallel fiber, *MF* mossy fiber. White, excitatory neurons; black, inhibitory neurons

Studies on LTDpathies might also lead to the assessment of dysregulated LTD in cerebellar cognitive affective syndrome [30]. Patients with anti-mGluR1 Ab-associated CA manifest with various behavioral changes (e.g., irritability, apathy, mood, personality change, psychosis with hallucinations, and catatonia) and cognitive changes (e.g., memory problems, executive functions, and spatial orientation deficits) [31]. Although these symptoms have been described as extra-cerebellar symptoms in the above report, the relation between these behavioral/cognitive changes with cerebellar cognitive affective syndrome should be reexamined carefully [30]. In the developing cerebellum, LTDpathies might cause synaptic punning-related symptoms such as autism spectrum disorder. There has been accumulating evidence showing that LTD and synaptic pruning share components of their underlying molecular machinery and LTD deficits can indicate impaired pruning processes required for proper brain development [32].

In conclusion, the concept of “LTDpathies” opens a new direction into LTD research and underlines the importance of recognizing these disorders in the clinic (Table 1). We anticipate that future translational studies at the level of the synapse or neural circuits will successfully provide a clear strategy for future therapies based on manipulations of critical molecules (cell designs) in order to relieve CAs. The identification of LTDpathies underlines the critical role of synaptic plasticity in the cerebellum.

**Table 1:** Clinical and physiological evidence reporting antibodies impaired long-term depression so as to develop cerebellar ataxias

Clinical evidence	• CAs associated with anti-VGCC, anti-mGluR1, and anti-GluR delta Abs show a common clinical features of good prognosis of the cerebellar symptoms. There is no or mild cerebellar atrophy on the brain MRI in non-paraneoplastic syndrome. All these data suggest that they share one common functional mechanism [13, 17]
Physiological evidence *in vitro*	• A polyclonal peptide Ab against the major immunogenic region in P/Q-type VGCCs (the extracellular domain-III S5-6 loop) impaired the functions of neuronal and recombinant P/Q-type VGCC [33] • The IgGs from the mGluR1 patients blocked the induction of LTD in tissue slices [23] • Ab against the H2 ligand binding site of GluR delta impaired induction of LTD in cultured PC [24]
Physiological evidence *in vivo*	• Application of IgGs from mGluR1 patients in the subarachnoid space elicited ataxic gaits in mice, which disappeared after absorption of anti-mGluR1 Ab [34], while administration of the same Ab in the flocculus also evoked acute disturbances in compensatory eye movements [23] • Subarachnoidal injection of Ab against the H2 ligand binding site of GluR delta elicited the development of ataxic symptoms in mice [24] • Acute blockade of mGluR was associated with impairment of PF-PC LTD and simultaneous motor incoordination without affecting basal synaptic transmission [25]

*CA* Cerebellar ataxia, *VGCC* Voltage-gated Ca channel, *mGluR1* Metabotropic glutamate receptor type 1, *GluR delta* Glutamate receptor delta, *Abs* Antibodies, *LTD* Long-term depression, *PC* Purkinje cell, *PF* Parallel fiber

## Data Availability

The concept reported in this manuscript is not associated with raw data.

## Abbreviations

Ab: Antibody
CF: Climbing fiber
GluR delta: Glutamic receptor delta
IMCAs: Immune-mediated cerebellar ataxias
LTD: Long-term synaptic depression
MF: Mossy fiber
mGluR1: Metabotropic glutamate receptor type 1
PC: Purkinje cell
PF: Parallel fiber
VGCC: Voltage-gated Ca channel
